# Evolving determinants of carotid atherosclerosis vulnerability in asymptomatic patients from the MAGNETIC observational study

**DOI:** 10.1038/s41598-021-81247-y

**Published:** 2021-01-27

**Authors:** Oronzo Catalano, Giulia Bendotti, Alessia Mori, Maria De Salvo, Marialuisa Falconi, Teresa L. Aloi, Valentina Tibollo, Riccardo Bellazzi, Alberto Ferrari Bardile, Stefano Montagna, Clara Pesarin, Paolo Poggi, Roberto F. E. Pedretti, Silvia G. Priori

**Affiliations:** 1 Division of Cardiology, Istituti Clinici Scientifici Maugeri, via Maugeri 6, Pavia, Italy; 2 Angiology Unit, Istituti Clinici Scientifici Maugeri, Pavia, Italy; 3 Bioinformatics Laboratory (LISRC Lab), Istituti Clinici Scientifici Maugeri, Pavia, Italy; 4 Department of Electrical, Computer and Biomedical Engineering, University of Pavia, Pavia, Italy; 5 Division of Radiology, Istituti Clinici Scientifici Maugeri, Pavia, Italy; 6 Molecular Cardiology, Istituti Clinici Scientifici Maugeri, Pavia, Italy; 7 University of Pavia, Pavia, Italy

**Keywords:** Cardiology, Diseases, Medical research, Pathogenesis, Risk factors

## Abstract

MRI can assess plaque composition and has demonstrated an association between some atherosclerotic risk factors (RF) and markers of plaque vulnerability in naive patients. We aimed at investigating this association in medically treated asymptomatic patients. This is a cross-sectional interim analysis (August 2013–September 2016) of a single center prospective study on carotid plaque vulnerability (MAGNETIC study). We recruited patients with asymptomatic carotid atherosclerosis (US stenosis > 30%, ECST criteria), receiving medical treatments at a tertiary cardiac rehabilitation. Atherosclerotic burden and plaque composition were quantified with 3.0 T MRI. The association between baseline characteristics and extent of lipid-rich necrotic core (LRNC), fibrous cap (CAP) and intraplaque hemorrhage (IPH) was studied with multiple regression analysis. We enrolled 260 patients (198 male, 76%) with median age of 71-y (interquartile range: 65–76). Patients were on antiplatelet therapy, ACE-inhibitors/angiotensin receptor blockers and statins (196–229, 75–88%). Median LDL-cholesterol was 78 mg/dl (59–106), blood pressure 130/70 mmHg (111–140/65–80), glycosylated hemoglobin 46 mmol/mol (39–51) and BMI 25 kg/m^2^ (23–28); moreover, 125 out of 187 (67%) patients were ex-smokers. Multivariate analysis of a data-set of 487 (94%) carotid arteries showed that a history of hypercholesterolemia, diabetes, hypertension or smoking did not correlate with LRNC, CAP or IPH. Conversely, maximum stenosis was the strongest independent predictor of LRNC, CAP and IPH (p < 0.001). MRI assessment of plaque composition in patients on treatment for asymptomatic carotid atherosclerosis shows no correlation between plaque vulnerability and the most well-controlled modifiable RF. Conversely, maximum stenosis exhibits a strong correlation with vulnerable features despite treatment.

## Introduction

Magnetic resonance imaging (MRI) provides a means to non-invasively assess luminal narrowing and composition of atherosclerotic carotid plaques. Many studies have correlated MRI findings with histology of excised carotid plaques, demonstrating that MRI can accurately assess lipid-rich necrotic core (LRNC), fibrous cap (CAP), intraplaque hemorrhage (IPH), calcification (CA) and plaque surface abnormalities^1–9^. Retrospective and prospective studies have shown an association between carotid plaques assessed by MRI and cerebral ischemic events^10–13^. Recently, observational studies have assessed carotid atherosclerosis features in asymptomatic and symptomatic patients and their correlations with risk factors (RF) for atherosclerosis^14–16^. In asymptomatic patients with carotid plaques, IPH and LRNC, two indexes of plaque vulnerability are highly frequent, occurring in about 25% of plaques and in about 75% of patients assessed with MRI^14^.

The MAGNETIC (Magnetic resonance imaging As a Gold standard for Noninvasive Evaluation of a Therosclerotic Involvement of Carotid arteries) study is an observational prospective study, on natural history of vulnerable carotid plaques, in a cohort of patients with asymptomatic mild to moderate carotid atherosclerosis, medically treated in a tertiary rehabilitation setting. Using serial multi-contrast MRI and a quantitative analysis of MRI images, the study is testing the hypothesis of the potential reversibility of plaque vulnerability features. The present work assessed the MAGNETIC study population characteristics at the baseline, with focus on plaque composition and atherosclerotic burden in order to confirm the correlation between RF and vulnerable plaque components.

## Results

The study enrolled 260 Caucasian patients from August 2013 to September 2016 (baseline characteristics are summarized in Table 1). Fourteen patients were excluded from the analysis due to poor image quality. Moreover, in five patients one carotid axis was excluded because of poor image quality, a high bifurcation position or excessive vessel angulation. In this interim analysis of baseline assessment, a dataset of 487 (94%) carotid arteries with adequate image quality was analyzed.

**Table 1:** Baseline characteristics of the study population (260 patients).

	Median (interquartile range)	n (%)
Sex (m)		198 (76)
**Age (years)**	71 (65–76)	
< 60		33 (13)
60–69		81 (31)
70–79		113 (43)
≥ 80		33 (13)
**Atherosclerosis risk factors**		
Familial history of premature CAD		51 (20)
Smoke		187 (72)
Active smoke		62 (24)
Hypercholesterolemia		168 (65)
Diabetes		92 (35)
Hypertension		207 (80)
Total # risk factors	4 (3–4)	
≥3 RF		212 (82)
BMI	25 (23–28)	
BMI ≥30 kg/m^2^		30 (12)
**Extra carotid atherosclerosis**		
Coronary artery disease		171 (66)
Peripheral arterial disease		52 (20)
**Blood chemistry**		
LDL Cholesterol (mg/dl)	78 (59–106)	
LDL Chol. ≥ 70 mg/dl		157 (60)
Triglycerides (mg/dl)	118 (92–169)	
Triglycerides ≥ 150 mg/dl		77 (30)
HDL Cholesterol (mg/dl)	43 (33–52)	
HDL ≤ 35 mg/dl		80 (31)
Glycosylated hemoglobin (mmol/mol)	43 (39–51)	
Glycosilate HB ≥ 54 mmol/mol		48 (18)
HS C-Reactive protein (mg/dl)	0.36 (0.13–1.24)	
HS-CRP ≥ 3 mg/dl		38 (1.5)
EGFR	68 (55–83)	
EGFR > 90, 60–89, < 60		41 (16), 128 (49), 91 (35)
Arterial blood pressure		
Systolic BP mmHg	130 (111–140)	
SBP ≥ 140 mmHg		69 (27)
Diastolic BP mmHg	70 (65–80)	
DBP ≥ 90 mmHg		6 (2)
**Medical therapy**		
Aspirin/antiplatelet		229 (88)
ACE-inhibitors/ARB		197 (76)
Statins		196 (75)

*CAD* coronary artery disease, *BMI* body mass index, *LDL* low density lipoprotein, *HDL* high density lipoprotein, *EGFR* estimated glomerular filtration rate, *BP* blood pressure, *ACE* angiotensin converting enzyme.

Two hundred twenty-nine patients (88%) were on aspirin/antiplatelet therapy, 197 (76%) on ACE-inhibitors/angiotensin receptor blocker and 196 (75%) on statins. Baseline median LDL cholesterol was 78 mg/dl (59–106), blood pressure 130/70 mmHg (111–140/65–80), glycosylated hemoglobin 46 mmol/mol (39–51) and BMI 25 (23–28).

Maximum stenosis was less than 50%, between 50 and 70% and more than 70% respectively in 19%, 67% and 14% of assessable carotid axes.

### Determinants of vulnerable plaque: univariate analysis

Univariate analysis did not show a significant association between most modifiable RF and components linked to plaque vulnerability. Indeed, no correlation of LRNC, CAP and IPH with a history of hypercholesterolemia, diabetes, hypertension and smoke, or with RF burden (total number of RF) was found, with the exception of a weak association of LRNC with RF burden (Spearman’s ρ = 0.09, p = 0.038). Moreover, there was no correlation between a composite score of baseline RF level and plaque vulnerability.

Carotid atherosclerosis metrics were linked to changes in atherosclerotic plaque composition. In fact, a moderate correlation was found between peak stenosis and LRNC, CAP and IPH (ρ between 0.35 and 0.53; p < 0.001). Similar results were found considering atherosclerotic burden (ρ between 0.31 and 0.42; p < 0.001).

Male sex was mildly correlated with LRNC (Mann–Whitney test, p = 0.007), CAP (p = 0.001) and IPH (p = 0.017), although men and women showed an identical burden of atherosclerotic RF (three [range: two to three] vs three [range: two to three]; p = 0.406). Men had higher carotid lumen volume (920 mm^3^ [745–1096] vs 774 mm^3^ [608–971); p < 0.001) and maximum area (48 mm^2^ [40–60] vs 38 mm^2^ [31–52]; p < 0.001).

There was a marginal correlation between age and LRNC (ρ = 0.10, p = 0.022) but not between age and CAP or IPH.

CAD comorbidity was mildly linked with LRNC (p < 0.001), CAP (p = 0.004) and IPH (p < 0.001). Familial history of premature CAD was mildly associated with LRNC (p = 0.008) as well.

PAD comorbidity was not associated with LRNC, CAP and IPH.

Because of the exclusion of severe/end stage chronic kidney disease (CKD), two-thirds of enrolled patients showed normal or near-normal eGFR (CKD stage 1–2: n = 169, 65%), while one-third moderate renal impairment (stage 3: n = 91, 35%). We found a mild inverse correlation between eGFR and LRNC (ρ = − 0.13; p = 0.005) and CAP (ρ =  − 0.10; p = 0.026) but no association between eGFR and IPH (ρ =  − 0.07; p = 0.099).

Left carotid side mildly correlated with LRNC (p = 0.013), CAP (p = 0.029) and IPH (p = 0.013).

**Figure 1:**
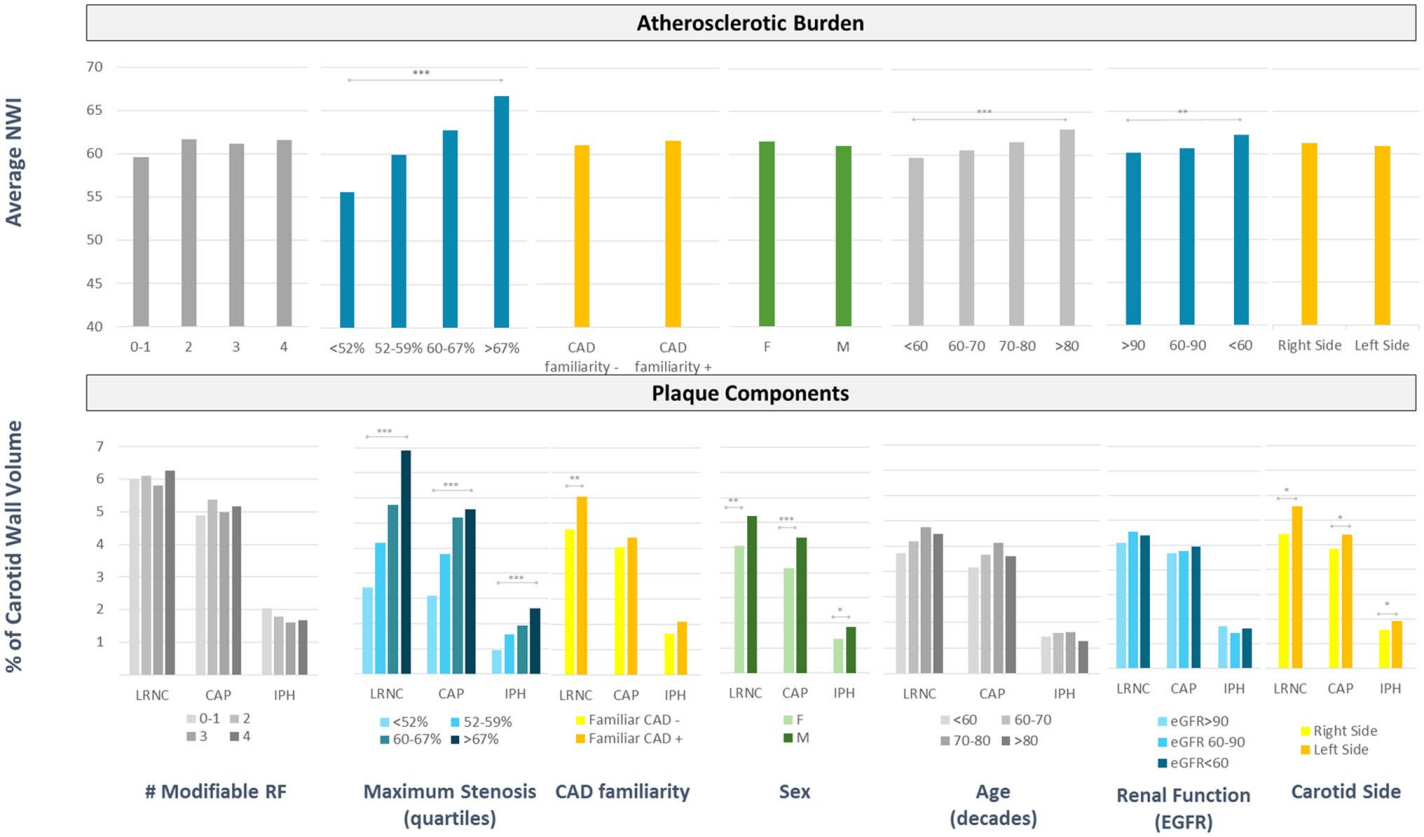
Trends of atherosclerotic burden and vulnerable plaque components by modifiable risk factors, maximum stenosis, familial history of premature CAD, sex, age, renal function and carotid side (univariate analysis). *NWI* normalized wall index, *CAD* coronary artery disease, *LRNC* lipid-reach necrotic core, *CAP* fibrous cap, *IPH* intraplaque hemorrhage, *RF* risk factors, *EGFR* estimated glomerular filtration rate. Bars are the median value. Kruskal–Wallis test was used to test differences, with Bonferroni correction for multiple comparisons; ***p < 0.001; **p < 0.01; *p < 0.05.

The complete set of univariate correlations and a graphical display of univariate association between plaque components (and atherosclerotic burden) and key baseline population characteristics are shown in Supplementary Table S2 and Fig. 1, respectively.

### Determinants of vulnerable plaque: multiple regression analysis

Multiple regression analysis (Table 2) showed that maximum stenosis was the strongest independent factor correlated with plaque vulnerability (association with LRNC, CAP and IPH: p < 0.001). Moreover, there was an independent association of features of plaque vulnerability with left side, male sex, familial history of premature CAD, coexisting CAD and BMI.

**Table 2:** Final multiple regression models predicting vulnerable plaque components.

	Model	B non-standardized coefficient (95% C.I.)	β standardized coefficient	t	p
Lipid-rich necrotic core	(Constant)	− 10.02 (− 12.78; − 7.26)		− 7.13	< 0.001
	Maximum stenosis	0.19 (0.17; 0.22)	0.50	13.15	< 0.001
	Left carotid side	1.10 (0.46; 1.74)	0.13	3.37	0.001
	Male sex	1.04 (0.28; 1.81)	0.10	2.67	0.008
	Fam. Hist. of CAD	1.01 (0.17; 1.85)	0.09	2.37	0.018
	Body mass index	0.07 (0.01; 0.15)	0.08	2.06	0.040
Fibrous cap	(Constant)	− 3.70 (− 5.39; − 2.00)		− 4.29	< 0.001
	Maximum stenosis	0.12 (0.10; 0.14)	0.40	9.86	< 0.001
	Male sex	1.09 (0.46; 1.71)	0.19	3.41	0.001
	Left carotid side	0.59 (0.06; 1.12)	0.09	2.19	0.029
Intraplaque hemorrhage	(Constant)	− 3.87 (− 5.40; − 2.33)		− 4.95	< 0.001
	Maximum stenosis	0.06 (0.04; 0.07)	0.30	6.87	< 0.001
	Body mass index	0.05 (0.01; 0.09)	0.11	2.59	0.010
	CAD comorbidity	0.44 (0.06; 0.83)	0.10	2.27	0.024
	Left carotid side	0.39 (0.03; 0.75)	0.09	2.13	0.034

This table shows only significant variables in the final model. The complete set of variables included in the analysis are reported in Supplementary Material.

*Fam. Hist. of CAD* familial history of coronary artery disease.

## Discussion

### Modifiable risk factors and markers of plaque vulnerability

In recent years, MRI has widened and improved assessment of atherosclerotic plaque composition, beyond other noninvasive imaging techniques like ultrasound and computer tomography.

With MRI, it has been possible to demonstrate that plaque vulnerability characteristics are related to atherosclerotic RF. This was found in naive populations with asymptomatic carotid atherosclerosis. Van den Bouwhuijsen et al. (the Rotterdam Study) showed a correlation between hypertension, current smoking and presence of IPH, and between hypercholesterolemia and LRNC^11^. In other cohorts of asymptomatic patients, Wasserman et al. (the MESA Study) and Virani et al. (the ARIC Study) showed that plasma LDL cholesterol and non-HDL cholesterol are the most important determinants of LRNC^15,16^.

In the present study, a baseline interim analysis of MAGNETIC prospective observational study, we investigated the persistence of a correlation between RF and plaque components in a cohort of asymptomatic subjects followed at a tertiary rehabilitation facility and receiving evidence-based medical treatment. In this setting, we found no association between MRI assessment of high-risk plaque features and a history of hypertension, diabetes mellitus, hypercholesterolemia and smoking, and only a mild association between BMI and intraplaque hemorrhage and lipid content. Thus, it seems that optimization of medical therapy and a healthier lifestyle might have blunted the association between modifiable RF and plaque vulnerability. These findings are consistent with recent results that showed, in biobanked carotid plaques, a significant decrease over time of large atheromas and an increase of plaques with fibrous non-inflammatory characteristics^22^. Our observations are also consistent with the progressive decrease in stroke incidence, observed since the mid-1980s, in the medical arms of randomized trials comparing different treatment strategies for asymptomatic severe carotid atherosclerosis^23^.

### Sex, familial predisposition and markers of plaque vulnerability

We also investigated the association between plaque vulnerability and non-modifiable genetically determined or potentially transmissible characteristics, such as male sex and familial history of premature CAD. As the effect of sex is concerned, it is well known that stroke incidence and prevalence is higher in men than in women between the ages of 45 and 74^24^. In addition, histological analysis of excised plaques^25,26^ and in vivo assessment with MRI^14,27^ have documented differences in plaque morphology in relation to sex. In agreement with these studies, we found that components associated with plaque vulnerability (IPH, LRNC and CAP) are quantitatively more represented in men than in women. This finding has been assumed to be related to variability in cardiovascular RF, levels of hormones or carotid anatomy^28^. In our cohort, atherosclerotic risk was globally balanced between the two sexes but men showed a larger lumen size than women. Thus, it might be hypothesized that hemodynamic and anatomic factors may be relevant for carotid plaque vulnerability in men. Notably, association between male sex and both LRNC and CAP remained unchanged after correction for all other significant factors.

As regards CAD comorbidity, it is known that the presence of carotid plaque predicts future CAD events, and that carotid and coronary vulnerable plaques share a similar pattern of disruption^29,30^. The correlation between vulnerable plaque components and the presence of CAD in the index patient or even in a first-degree relative, found in the present study, seems to confirm a common substrate potentially able to trigger plaque vulnerability and, as a consequence, a coronary or a cerebral ischemic event. Multiple regression analysis showed that CAD comorbidity and familial history of premature CAD are independently associated with plaque vulnerability.

### Carotid artery aging, atherosclerotic burden and plaque composition

Atherosclerosis may develop at an early age and increase with aging^31^. In a cohort of subjects with asymptomatic carotid atherosclerosis, we observed that age correlates with carotid wall volume, maximum stenosis and atherosclerotic burden. However, we found only a mild association between age and LRNC content, and no association with other markers of plaque vulnerability, such as IPH. This is in contrast with previous studies^14,32^. Significant lower blood pressure in our cohort might have blunted the confounding effect of this factor, known to be associated with both aging and IPH. Like aging, renal function impairment correlated with modifications of carotid wall, such as increase of wall volume and atherosclerotic burden, and with LRNC content. Neither age nor eGFR were found to be independently associated with plaque vulnerability in the present study.

### Luminal stenosis, atherosclerotic burden and plaque composition

The degree of carotid luminal stenosis is one of the main criteria for identifying high-risk asymptomatic patients who could benefit from carotid endarterectomy^33^. Studies from the 1980’s and 1990’s demonstrated an association of IPH and plaque ulceration with the degree of stenosis. Recent MRI studies have also shown a positive correlation between the presence of high-risk features (LRNC, IPH and CAP) and an increasing degree of stenosis, maximum wall thickness or plaque burden^15,34,35^. Our study confirms the persistence of an association between severity of carotid atherosclerosis and plaque vulnerability in a cohort of patients compliant with secondary prevention measures.

### Carotid laterality as an additional element of risk

Previous studies showed that extracranial carotid-artery disease is responsible for up to 20% of strokes and that one-third of patients with cryptogenic stroke have ipsilateral non-stenotic or mildly stenotic carotid plaques^36^.

We found no difference in maximum stenosis or atherosclerotic burden between the right and the left carotid artery. However, we observed a higher incidence of high-risk plaque features (LRNC, CAP and IPH) on the left side, as also confirmed with multiple regression analysis. To the best of our knowledge, this is the first study reporting differences in plaque composition between the right and left carotid. This finding could explain the higher rate of ischemic stroke in the left cerebral hemisphere, as shown in previous studies^37,38^. Side-related differences in plaque composition could be due to a different shear stress between left and right side. Indeed, there is evidence that low shear stress causes endothelial cell dysfunction, increases lipoproteins uptake and stimulates plaque development^39,40^. No study so far has demonstrated differences in shear stress between the carotid sides. However, it is known that shear stress varies inversely with vessel diameter and in the present study the left carotid artery showed a higher maximum lumen area (p < 0.01) compared to the right carotid. Accordingly, localized reduction of shear stress might influence the observed side differences in plaque composition.

### Limits of the study

Due to its cross-sectional nature, this study cannot assert that lack of association between plaque vulnerability and modifiable risk factors is the effect of treatment of risk factors themselves, albeit such an effect is biologically plausible. Moreover, we did not record at the enrollment how long patients have been receiving treatment on atherosclerosis. This information could be helpful for a better understanding of the impact of medical therapy and a healthier lifestyle on plaque vulnerability and should be tested in a prospective study.

A surplus of men was not intentional but the result of referral population composition. Generalization of the results of the study must consider this datum.

This is a single center study. Its findings need to be confirmed by other researches.

### Conclusion

In patients with asymptomatic carotid atherosclerosis receiving evidence-based medical treatments, quantification of plaque components with high-resolution MRI demonstrates a blunted role of well-controlled modifiable RF (hypercholesterolemia, hypertension, diabetes, smoke) on the determination of a vulnerable plaque composition. Despite treatment, maximum lumen stenosis seems to be the most important independent predictor of plaque vulnerability.

## Methods

### Study design

The MAGNETIC study is a single center prospective observational study: it includes subjects with ultrasound evidence of carotid atherosclerosis, consecutively evaluated in a cardiovascular rehabilitation facility. The study was completed in 2019. Its primary endpoint is the reversibility of plaque vulnerability. The research is designed to detect, with α equal to 5% and power equal to 80%, a regression from a vulnerable state in 30% of subjects defined at high risk at the study start. The study design, criteria defining plaque vulnerability and additional information about sample size calculation, are provided as supplementary material (Supplementary Fig. S1, Supplementary Table S1, Supplementary File 1).

The Ethical Committee of Istituti Clinici Scientifici Maugeri (Pavia, Italy) approved the study and all patients gave informed consent to participate in it. All methods were carried out in accordance with relevant guidelines and regulations.

Inclusion criteria were: age > 45 years; echographic evidence of 30–70% carotid stenosis, or more severe stenosis in cases of refused or contraindicated surgery. Exclusion criteria were: prior cerebral ischemia or carotid intervention (endarterectomy or stenting); contraindications for high-field MRI, claustrophobia or dementia; estimated glomerular filtration rate (eGFR) < 30 ml/min/1.73 m^2^; allergic diathesis to gadolinium or any excipients of the contrast media; pregnancy or breast-feeding; participating in another study; any life-threatening disease with a life expectancy of less than 1 year.

The baseline cross-sectional phase of the study (completed in September 2016), included medical examination, ECG, carotid MRI and blood chemistry (high sensitive C-reactive protein, LDL cholesterol, HDL cholesterol, triglycerides, glycosylated hemoglobin, urea and creatinine), obtained on the same day. Clinical evaluation included a neurologic assessment. Patients were excluded from the study if they had a history of previous symptomatic cerebral ischemia or National Institutes of Health Stroke Scale (NIHSS) score greater than 2. Anthropometric data, atherosclerotic RF profile (according to the current European Society of Cardiology guidelines), documented history of coronary artery disease (CAD) or peripheral arterial disease (PAD) and pharmacological therapy were also recorded.

### Ultrasound imaging

The maximum vessel diameter stenosis (peak stenosis) along the common/internal carotid artery (CCA, ICA) was calculated by senior vascular specialists using an up-to-date scanner (MyLab ClassC, Esaote S.p.A.), in accordance to the European Carotid Surgery Trial (ECST) method.

### MRI protocol

Baseline carotid MRI was performed on a 3.0 T MRI scanner (Discovery MR750, GE Healthcare) with a bilateral six channels phased-array surface coil. Carotid bifurcations were identified by means of 3D time-of-flight (TOF) MR angiography. Thereafter, carotid bifurcations were imaged with the following MRI sequences: 2D T1-weighted, PD-weighted, and T2-weighted black-blood images and 3D TOF bright-blood angiography (FOV 16 cm, matrix size 256 × 256, in-plane resolution 0.62 × 0.62 mm, 2 signal-intensity averages). T1-weighted sequence was repeated 5 min after the administration of gadolinium contrast (Gadobutrol 1.0 mmol/ml, 0.1 mmol/kg—Bayer AG, Leverkusen). Sixteen 2-mm thick contiguous sections perpendicular or quasi-perpendicular (max tolerance: 15°) to CCA/ICA long axis were acquired. Total coverage was 32 mm around the carotid bifurcation. No adverse events occurred with injection of contrast medium.

### Images analysis

**Figure 2:**
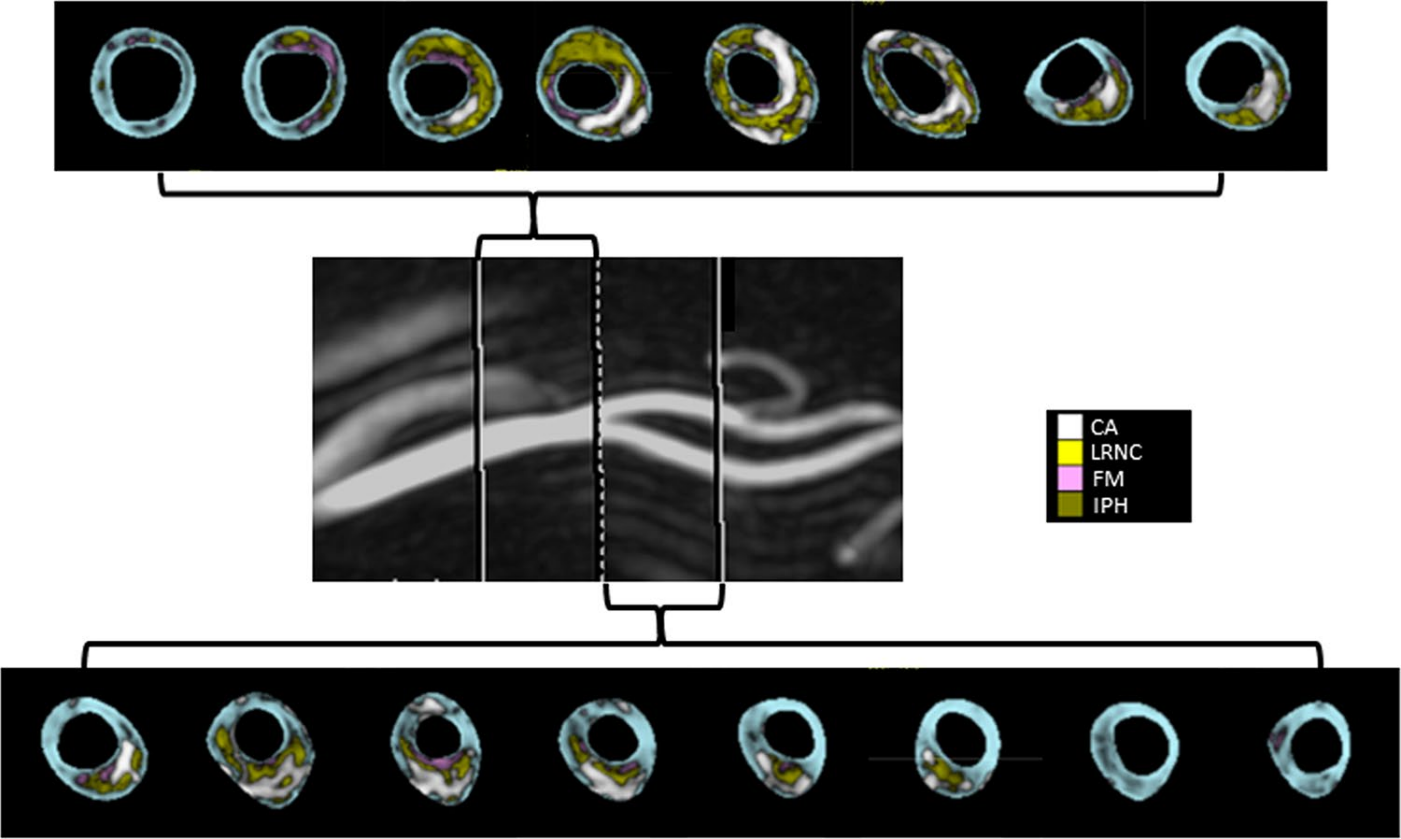
Processed images of a carotid axis. Color coded areas identify different plaque components. *CA* calcification (white), *LRNC* lipid reach necrotic core (yellow), *FM* fibrous matrix (pink), and *IPH* intraplaque hemorrhage (green). The layer of tissue separating a LRNC area from the lumen is considered fibrous cap.

Images with a quality score greater than 2, on a 5-point scale from poor to optimal (mark from 1 to 5) were post-processed. Quality score was an average value between a mark assigned at the scanning time by an experienced radiologist, based on image contrast-to-noise ratio and artifacts, and a score assigned at post-processing time, by a cardiologist with a specific training in the use of post-processing software, mainly relying on the amount of manual correction needed to draw wall and lumen contour. A validated software (MRI-Plaque View, VPDiagnostic, Seattle)^8,17–21^, was used to analyze carotid wall composition, through semi-automatic slice-to-slice contouring of internal and external vessel borders (CCA and ICA), and multisequence signal comparison (an example of color- coded processed images is shown in Fig. 2). Metrics of carotid atherosclerosis were: peak stenosis along the CCA or the ICA (ESCT method); atherosclerotic burden, intended as the average normalized wall index (NWI = wall area/total vessel area × 100%); and LRNC, CAP and IPH percentage of the wall volume (CCA plus ICA).

### Statistics

SPSS 17.0 software was used. Categorical variables were expressed as counts and percentage, continuous variables as median and interquartile range. Univariate associations were tested with Spearman’s ρ coefficient or Mann–Whitney test. Least square multiple regression analysis, considering variables with p ≤ 0.10 at univariate tests, was used to assess independent association between plaque components and cohort’s baseline characteristics. Two-sided tests and a significance level < 0.05 were used for hypothesis testing.

## Supplementary Information

Supplementary Information.
