# Supplementary material for: Evolving determinants of carotid atherosclerosis vulnerability in asymptomatic patients from the MAGNETIC observational study
Source: Sci Rep. 2021 Jan 27;11:2327. doi: 10.1038/s41598-021-81247-y (PMC7840938; doi:10.1038/s41598-021-81247-y)
Supplement: Supplementary file 1 — Supplementary Information. [file 41598_2021_81247_MOESM1_ESM.docx]

Evolving determinants of carotid atherosclerosis vulnerability in asymptomatic patients from the MAGNETIC observational study

^1 *^Oronzo Catalano, MD;  ^1^ Giulia Bendotti, MD; ^1^ Alessia Mori, MD; ^1^ Maria De Salvo, MD; ^1^ Marialuisa Falconi; RN; ^2^ Teresa L. Aloi, MD; ^3^ Valentina Tibollo, Eng; ^4^ Riccardo Bellazzi; PhD, Prof; ^2^ Alberto Ferrari Bardile, MD; ^5^ Stefano Montagna, RT; ^5^ Clara Pesarin, RT; ^5^ Paolo Poggi, MD; ^1^ Roberto F.E. Pedretti, MD and  ^6^ Silvia G. Priori, MD, PhD, Prof

^1^ Division of Cardiology, Istituti Clinici Scientifici Maugeri, Pavia, Italy;

^2^ Angiology Unit, Istituti Clinici Scientifici Maugeri, Pavia, Italy;

^3^ Bioinformatics Laboratory (LISRC Lab), Istituti Clinici Scientifici Maugeri, Pavia, Italy;

^4^ Department of Electrical, Computer and Biomedical Engineering, University of Pavia, Pavia, Italy

^5^ Division of Radiology, Istituti Clinici Scientifici Maugeri, Pavia, Italy;

^6^ Molecular Cardiology, Istituti Clinici Scientifici Maugeri; and University of Pavia; Pavia, Italy;

SUPPLEMENTARY METHODS

Study power and sample size . We made two hypotheses of change state, at patient level: we hypothesized a pessimistic scenario with 10% and an optimistic scenario with 50% conversion rate (mean rate: 30%) from high risk to intermediate/low risk during the 3-year follow-up period. Assuming confidence intervals equal to ± 10%, α equal to 5% and power equal to 80%, sample size was 96 patients in the pessimistic and 35 patients in the optimistic scenario, respectively. Under the assumption that only part of subjects with evidence of LRNC and/or IPH have plaques at high risk (⅓ out of the 75% found in the Rotterdam study[^1^](https://paperpile.com/c/qaENRw/V9C7), equal to 25%), we forecasted that about 260 patients must be screened in order to enroll 65 patients at high risk (mean value between 35 and 96). Accordingly, 260 patients were enrolled from August 2013 to September 2016, at the ultrasound vascular lab of our cardiac rehab facility; in the same period 6423 carotid exams were performed, with 1313 potentially eligible subjects.

The main reason why many screened patients were not enrolled in the study was their will not to participate in the research.

In order to define carotid plaques vulnerability, according to previous studies, we considered LRNC, IPH, and CAP characteristics. Since quantitative cut-offs of components associated with plaque instability are still lacking, we applied a statistical approach to derive cut-off values for risk stratification.

Cut-off values derivation. A statistical approach was used to derive cut-off values. We analyzed, at the slice level, data of the first 20 patients enrolled in the study.  From the resulting 640 slices (20 pts x 2 carotid axes x 16 slices) we excluded those with minor atherosclerotic involvement, i.e. with stenosis of less than 30% (ESCT method). The remaining 346 slices were pooled together and the 95th percentiles of each plaque component, or the 5th percentile where appropriate, were calculated, obtaining the following cut-off values: i) LRNC area: 26% of vessel area, ii) IPH area: 12% of vessel area, iii) minimum CAP thickness: 0,06 mm; mean CAP thickness: 0,4 mm; CAP projection length: 11 mm.
Risk classification. An atherosclerotic plaque was considered at high risk if one of the following cases occurred: i) LRNC criterion fulfilled (stenosis >30% and LRNC >26% of vessel area) in two or more contiguous slices, ii) IPH criterion fulfilled (stenosis >30% and IPH >12% of vessel area) in two or more contiguous slices, iii) CAP criterion fulfilled (any two among minimum thickness < 0.06 mm, mean thickness < 0.4 mm and projection length > 11 mm) in two or more contiguous slices, iv) composite criterion fulfilled (any two of LRNC, IPH or CAP criteria at the same slice level). A carotid axis was considered at high risk if one or more plaques at high risk were present. A patient was considered at high risk if one or both carotid axes were at high risk. An atherosclerotic plaque was considered at intermediate risk if at least one among LRNC, IPH and CAP criteria was present in a single slice or in multiple non contiguous slices. A carotid axis was considered at intermediate risk if one or more plaques at intermediate risk were present. A patient was considered at intermediate risk if one or both carotid axes were at intermediate risk. The remaining patients were considered at low risk. Supplementary table 1 summarizes criteria defining carotid axis and patients at low, intermediate and high risk.

Variables included in multiple regression analysis.

The following variables (p≤0.10 at univariate tests) were included in multiple regression analysis to assess independent correlation with vulnerable plaque components:

- lipid-rich necrotic core: maximum stenosis, average normalized wall index (NWI), sex, age, familial history of CAD, smoke, # risk factors, CAD comorbidity, HDL cholesterol, BMI, EGFR, carotid side;
- fibrous cap: maximum stenosis, average NWI, sex, age, # risk factors, CAD comorbidity, systolic blood pressure, BMI, EGFR, carotid side;
- intraplaque hemorrhage: maximum stenosis, average NWI, sex, CAD comorbidity, LDL cholesterol, BMI, EGFR, carotid side.

1. [van den Bouwhuijsen QJA, Vernooij MW, Hofman A, Krestin GP, van der Lugt A, Witteman JCM. Determinants of magnetic resonance imaging detected carotid plaque components: the Rotterdam Study. *Eur Heart J*. 2011;33:221–229.](http://paperpile.com/b/qaENRw/V9C7)


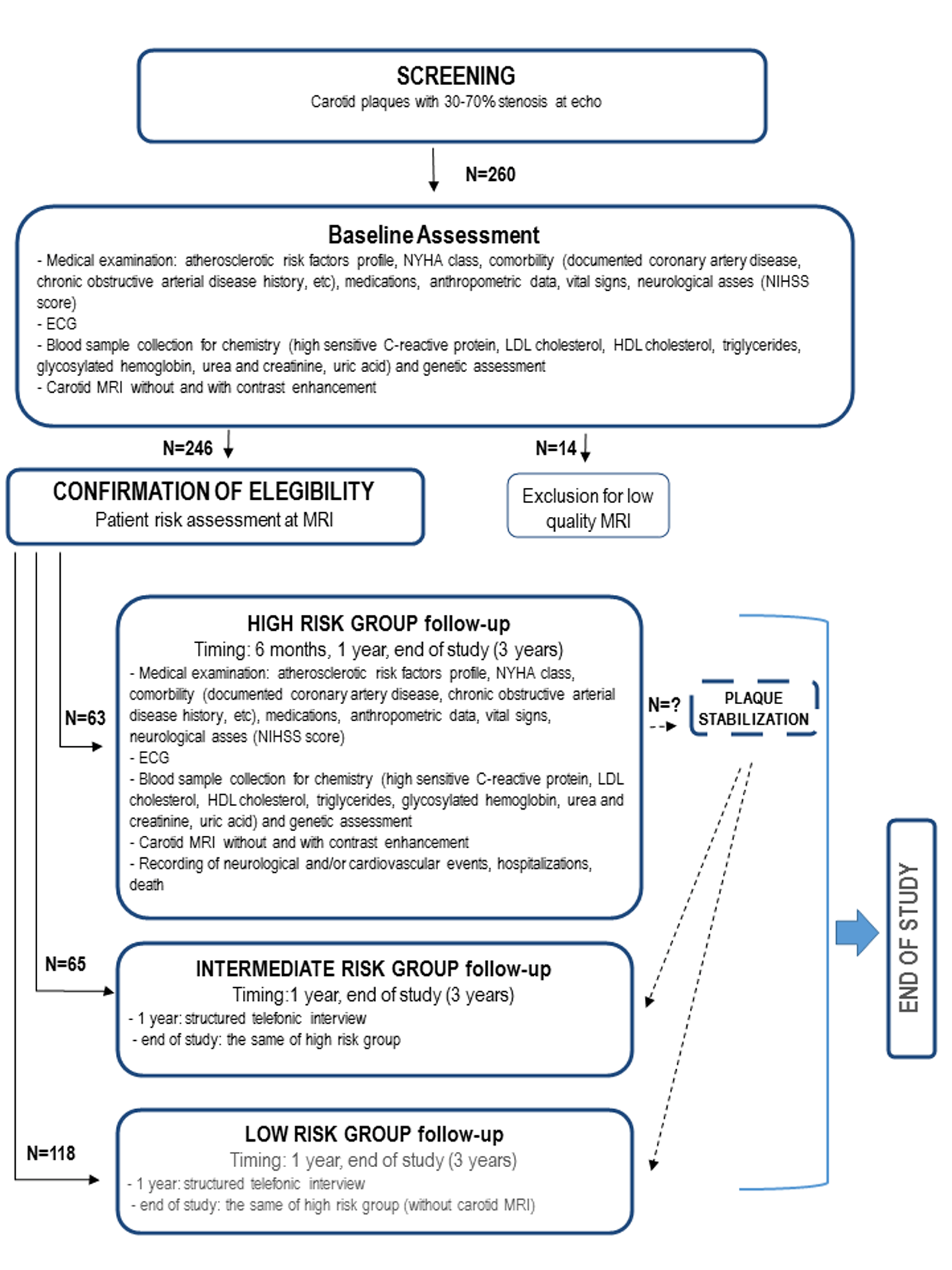


**Supplementary figure 1.** Study flow-chart diagram. Figure was created with Microsoft PowerPoint (www.office.com).

| **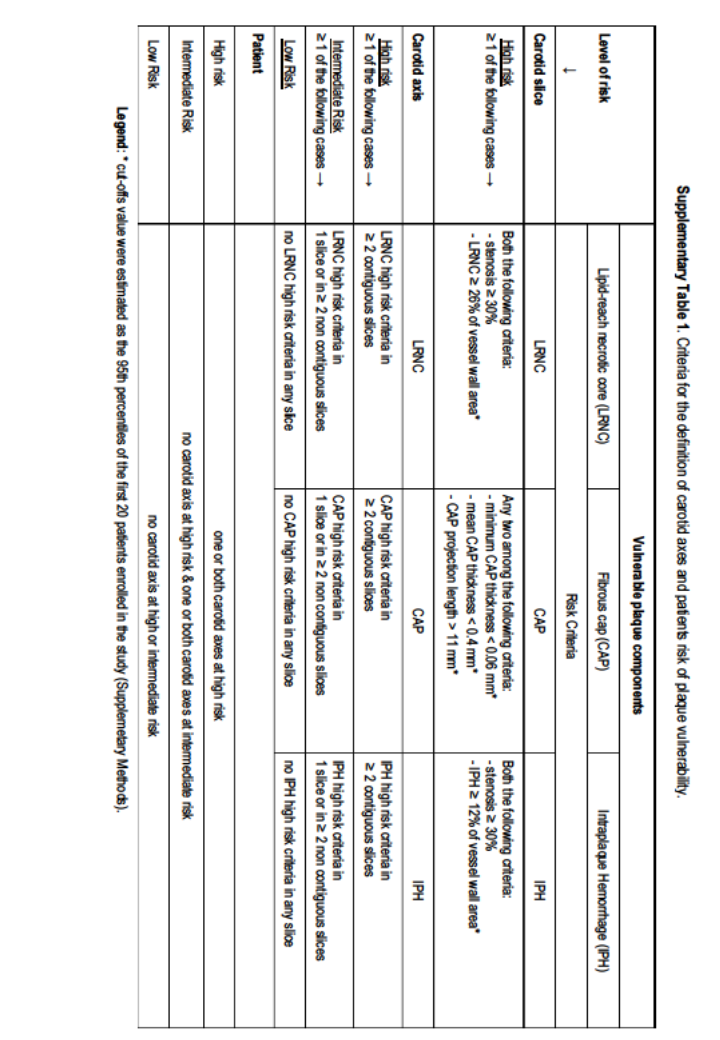** | | | | | | | | | |
| --- | --- | --- | --- | --- | --- | --- | --- | --- | --- |
| **Supplementary Table 2.**  Correlation analysis between vulnerable plaque components and baseline patient  characteristics. | | | | | | | | | |
|  |  | **Maximum Stenosis (%)** | **Average Normalized WI (%)** | **Lipid-Rich**  **Necrotic Core**  **(%)** | **Fibrous Cap**  **(%)** | **IntraPlaque Hemorrhage (%)** | |  |  |
| **Sex** | Mann-Whitney |  |  |  |  |  | | |  |
| (female=0;male=1) | p value | 0.304 | 0.632 | 0.007 | 0.001 | 0.017 | | |  |
|  |  |  |  |  |  |  | | |  |
| **Age** | Spearman’s ρ | 0.20 | 0.19 | 0.10 | 0.07 | 0.03 | | |  |
| (years) | p value | <0.001 | <0.001 | 0.022 | 0.099 | 0.457 | | |  |
|  |  |  |  |  |  |  | | |  |
| **Famil.Hist.of CAD** | Mann-Whitney |  |  |  |  |  | | |  |
| (no=0; yes=1) | p value | 0.348 | 0.313 | 0.008 | 0.124 | 0.218 | | |  |
|  |  |  |  |  |  |  | | |  |
| **Smoke** | Mann-Whitney |  |  |  |  |  | | |  |
| (no=0; yes=1) | p value | 0.505 | 0.233 | 0.096 | 0.138 | 0.134 | | |  |
|  |  |  |  |  |  |  | | |  |
| **Active Smoke** | Mann-Whitney |  |  |  |  |  | | |  |
| (no=0; yes=1) | p value | 0.543 | 0.512 | 0.399 | 0.619 | 0.324 | | |  |
|  |  |  |  |  |  |  | | |  |
| **Hypercholesterolemia** | Mann-Whitney |  |  |  |  |  | | |  |
| (no=0; yes=1) | p value | 0.278 | 1.000 | 0.432 | 0.701 | 0.356 | | |  |
|  |  |  |  |  |  |  | | |  |
| **Diabetes** | Mann-Whitney |  |  |  |  |  | | |  |
| (no=0; yes=1) | p value | 0.698 | 0.443 | 0.480 | 0.266 | 0.183 | | |  |
|  |  |  |  |  |  |  | | |  |
| **Hypertension** | Mann-Whitney |  |  |  |  |  | | |  |
| (no=0; yes=1) | p value | 0.509 | 0.980 | 0.811 | 0.686 | 0.756 | | |  |
|  |  |  |  |  |  |  | | |  |
| **# Risk Factors** | Spearman’s ρ | 0.04 | 0.03 | 0.09 | 0.08 | 0.05 | | |  |
|  | p value | 0.353 | 0.445 | 0.038 | 0.092 | 0.258 | | |  |
|  |  |  |  |  |  |  | | |  |
| **CAD** | Mann-Whitney |  |  |  |  |  | | |  |
| (no=0; yes=1) | p value | <0.001 | <0.001 | <0.001 | 0.004 | <0.001 | | |  |
|  |  |  |  |  |  |  | | |  |
| **PAD** | Mann-Whitney |  |  |  |  |  | | |  |
| (no=0; yes=1) | p value | 0.086 | 0.020 | 0.711 | 0.876 | 0.897 | | |  |
|  |  |  |  |  |  |  | | |  |
| **LDL Cholesterol** | Spearman’s ρ | -0.07 | -0.10 | -0.06 | -0.05 | -0.08 | | |  |
| (mg/dl) | p value | 0.130 | 0.027 | 0.186 | 0.228 | 0.066 | | |  |
|  |  |  |  |  |  |  | | |  |
| **HDL Cholesterol** | Spearman’s ρ | -0.05 | -0.05 | -0.08 | -0.03 | -0.05 | | |  |
| (mg/dl) | p value | 0.233 | 0.292 | 0.083 | 0.466 | 0.238 | | |  |
|  |  |  |  |  |  |  | | |  |
| **Triglycerides** | Spearman’s ρ | 0.01 | -0.03 | -0.02 | 0.02 | -0.06 | | |  |
| (mg/dl) | p value | 0.835 | 0.521 | 0.736 | 0.656 | 0.203 | | |  |
|  |  |  |  |  |  |  | | |  |
| **Systolic BP** | Spearman’s ρ | -0.03 | 0.02 | -0.04 | -0.09 | -0.05 | | |  |
| (mmHg) | p value | 0.457 | 0.718 | 0.354 | 0.044 | 0.269 | | |  |
|  |  |  |  |  |  |  | | |  |
| **Diastolic BP** | Spearman’s ρ | -0.07 | -0.04 | -0.02 | -0.01 | -0.07 | | |  |
| (mmHg) | p value | 0.121 | 0.362 | 0.662 | 0.877 | 0.134 | | |  |
|  |  |  |  |  |  |  | | |  |
| **Body Mass Index** | Spearman’s ρ | -0.07 | -0.03 | 0.09 | 0.09 | 0.15 | | |  |
| (kg/m^2^) | p value | 0.147 | 0.464 | 0.028 | 0.038 | 0.001 | | |  |
|  |  |  |  |  |  |  | | |  |
| **Glycosylated HB** | Spearman’s ρ | -0.04 | 0.04 | -0.02 | -0.07 | -0.05 | | |  |
| (mmol/mol) | p value | 0.425 | 0.365 | 0.581 | 0.144 | 0.313 | | |  |
|  |  |  |  |  |  |  | | |  |
| **HS-RCP** | Spearman’s ρ | 0.01 | 0.03 | 0.07 | 0.04 | 0.07 | | |  |
| (mg/dl) | p value | 0.762 | 0.438 | 0.118 | 0.416 | 0.118 | | |  |
|  |  |  |  |  |  |  | | |  |
| **EGFR** | Spearman’s ρ | -0.10 | -0.14 | -0.13 | -0.10 | -0.07 | | |  |
| (ml/min*1.73m^2^) | p value | 0.023 | 0.002 | 0.005 | 0.026 | 0.099 | | |  |
|  |  |  |  |  |  |  | | |  |
| **Composite baseline** | Spearman’s ρ | -0.02 | 0.03 | 0.07 | 0.01 | 0.06 | | |  |
| **RF level score** | p value | 0.717 | 0.523 | 0.153 | 0.918 | 0.162 | | |  |
|  |  |  |  |  |  |  | | |  |
| **Carotid Side** | Mann-Whitney |  |  |  |  |  | | |  |
| (right=0; left=1) | p value | 0.957 | 0.396 | 0.013 | 0.029 | 0.013 | | |  |
| **Legend**: Fam.Hist.of CAD=familial history of coronary artery disease; PAD= peripheral arterial disease; LDL=low density lipoprotein; HDL= low density lipoprotein; BP=blood pressure; HB=hemoglobin; HS-RCP=high sensitive C-reactive protein; EGFR=estimated glomerular filtration rate. # Risk is a score based on previous diagnosis of diabetes, hypertension, hypercholesterolemia and the presence of a familial history of CAD or of active smoke (one point for each RF; score range from 0 to 5). Composite baseline RF level score is a score calculated by assigning a progressively higher mark, from 1 to 4, according to increasing quartiles of glycosylated haemoglobin, pulse pressure, LDL cholesterol, triglycerides, BMI and PCR, or of worse smoke status (1 for non smokers, 2 for ex smokers >3 years, 3 for ex smokers within 3 years and 4 for active smokers) and a progressively lower score, from 4 to 1, for increasing quartiles for HDL cholesterol and eGFR (score range: from 9 to 36) | | | | | | |  | |  |
|  | | | | | | | | | |
